# Liver dysfunction associated with artificial nutrition in critically ill patients

**DOI:** 10.1186/cc5670

**Published:** 2007-01-25

**Authors:** Teodoro Grau, Alfonso Bonet, Mercedes Rubio, Dolores Mateo, Mercé Farré, José Antonio Acosta, Antonio Blesa, Juan Carlos Montejo, Abelardo García de Lorenzo, Alfonso Mesejo, the Working Group on Nutrition and Metabolism of the Spanish Society of Critical Care

**Affiliations:** 1 Intensive Care Unit, Hospital Severo Ochoa. Av. Orellana s/n, 28911 Leganés, Madrid, Spain; 2 Intensive Care Unit, Hospital Josep Trueta. Av. de Francia s/n, 17007 Girona, Spain; 3 Cardiovascular Intensive Care Unit, Hospital Universitario 12 de Octubre. Av. de Córdoba s/n, 28041 Madrid, Spain; 4 Intensive Care Unit, Newham University Hospital NHS Trust. Glen Road, Plaistow London E13 8SL, UK; 5 Intensive Care Unit, Hospital Universitari Vall d'Hebró. Paseo Vall d'Hebró 119-129, 08035 Barcelona, Spain; 6 Intensive Care Unit, General de Alicante. Maestro Alonso 109, 03010 Alicante, Spain; 7 Intensive Care Unit, Hospital Clínico San Carlos. Profesor Martin Lagos s/n, 28040 Madrid, Spain; 8 Intensive Care Unit, Hospital Universitario Doce de Octubre.Av. de Córdoba s/n, 28041 Madrid, Spain; 9 Intensive Care Unit, Hospital Universitario La Paz. Paseo de la Castellana 261, 28046 Madrid, Spain; 10 Intensive Care Unit, Hospital Universitario La Fe. Av. Campanar 21, 46009 Valencia, Spain

## Abstract

**Introduction:**

Liver dysfunction associated with artificial nutrition in critically ill patients is a complication that seems to be frequent, but it has not been assessed previously in a large cohort of critically ill patients.

**Methods:**

We conducted a prospective cohort study of incidence in 40 intensive care units. Different liver dysfunction patterns were defined: (a) cholestasis: alkaline phosphatase of more than 280 IU/l, gamma-glutamyl-transferase of more than 50 IU/l, or bilirubin of more than 1.2 mg/dl; (b) liver necrosis: aspartate aminotransferase of more than 40 IU/l or alanine aminotransferase of more than 42 IU/l, plus bilirubin of more than 1.2 mg/dl or international normalized ratio of more than 1.4; and (c) mixed pattern: alkaline phosphatase of more than 280 IU/l or gamma-glutamyl-transferase of more than 50 IU/l, plus aspartate aminotransferase of more than 40 IU/l or alanine aminotransferase of more than 42 IU/l.

**Results:**

Seven hundred and twenty-five of 3,409 patients received artificial nutrition: 303 received total parenteral nutrition (TPN) and 422 received enteral nutrition (EN). Twenty-three percent of patients developed liver dysfunction: 30% in the TPN group and 18% in the EN group. The univariate analysis showed an association between liver dysfunction and TPN (*p *< 0.001), Multiple Organ Dysfunction Score on admission (*p *< 0.001), sepsis (*p *< 0.001), early use of artificial nutrition (*p *< 0.03), and malnutrition (*p *< 0.01). In the multivariate analysis, liver dysfunction was associated with TPN (*p *< 0.001), sepsis (*p *< 0.02), early use of artificial nutrition (*p *< 0.03), and calculated energy requirements of more than 25 kcal/kg per day (*p *< 0.05).

**Conclusion:**

TPN, sepsis, and excessive calculated energy requirements appear as risk factors for developing liver dysfunction. Septic critically ill patients should not be fed with excessive caloric amounts, particularly when TPN is employed. Administering artificial nutrition in the first 24 hours after admission seems to have a protective effect.

## Introduction

Artificial nutrition support is part of the standard of care in critically ill patients [1]. Some of these patients have sepsis or systemic inflammatory response syndrome, which produce hypermetabolism, accelerated lipolysis, insulin resistance, and protein catabolism. These phenomena, associated with the lack of oral intake, can lead to malnutrition. Artificial nutrition usually does not reverse these metabolic derangements but can decrease the depletion of the lean body mass [2]. Hepatobiliary complications related to artificial nutrition have been widely reported, particularly in patients receiving total parenteral nutrition (TPN), and less frequently in patients receiving enteral nutrition (EN) [3]. There are many potential causes of liver dysfunction (LD) related to artificial nutrition, but the etiology is unclear and there are few data on the prevalence in critically ill patients. Moreover, these patients can present hepatic dysfunction as part of the multiple organ failure syndrome [4]. The aim of this study was to assess the prevalence of hepatobiliary complications related to artificial nutrition, the risk factors associated with these complications, and their influence on the prognosis in critically ill patients.

## Materials and methods

### Design

This study was designed as a multicenter prospective cohort study of incidence of LD in patients admitted to any of the 40 participating intensive care units (ICUs) from tertiary hospitals in Spain between 1 March and 15 April 2000. Patients were enrolled consecutively when the treating physician expected them to need artificial nutrition for five days or more. The protocol and definitions of LD were established previously in a meeting with the participants. The institutional review board of each participating hospital approved the study. Informed consent was waived according to these boards and Spanish law. Our funding sources had no role in the acquisition, analysis, or interpretation of data or in the submission of this report.

### Patients

Patients entered in the study were followed prospectively until hospital discharge or 28 days after ICU admission to check mortality at that time. Age, gender, weight, primary diagnosis, group (medical, surgical, or trauma), APACHE II (Acute Physiology and Chronic Health Evaluation II) score [5], Multiple Organ Dysfunction Score (MODS) [4], the need for mechanical ventilation, and the presence and origin of sepsis and/or septic shock were recorded on admission. The diagnosis of sepsis or septic shock on admission was made according to previously published criteria [6]. Sepsis was defined when a patient had a confirmed infection with two or more of the following criteria: (a) temperature greater than 38°C or less than 36°C, (b) heart rate greater than 90 beats per minute, (c) respiratory rate greater than 20 respirations per minute or PaCO_2 _(partial pressure of carbon dioxide) less than 32 mm Hg, and (d) leukocytes greater than 12,000 per cubic millimeter or greater than 10% band neutrophils. Septic shock was defined as arterial hypotension induced by sepsis, which persists in spite of the adequate replacement of fluids and associated with hypoperfusion and organ dysfunction. Exclusion criteria were age of less than 18 years, expected survival of less than 24 hours, or previous cardiopulmonary resuscitation. Patients with previously recognized liver disease were excluded by the following criteria: (a) portal hypertension with gastrointestinal bleeding at the time of admission and/or transfer, (b) clinically apparent ascites on a hepatocellular basis, (c) total bilirubin of more than 3 mg/dl or aspartate aminotransferase of more than 40 IU/l or on a hepatocellular basis, (d) serum albumin of less than 0.03 g/l with portal hypertension, (e) encephalopathy of grade II or greater, and (f) clinical diagnosis of alcoholic hepatitis [7].

### Choice of the type of nutrition

The clinician responsible for the patient chose the type of nutrition, the administration route, and the type of diet following the published recommendations [8]. The protocol was discussed in previous meetings with the researchers. The use of early artificial nutrition was encouraged to the participants. EN was recommended as the preferred route for feedings if the patient's gastrointestinal system was preserved. Clinicians could switch to TPN if the patient did not tolerate EN due to gastrointestinal complications or if 75% of the caloric requirements were not achieved after three days of enteral feedings. Also, clinicians were allowed to administer EN for as long as the gastrointestinal function was recovered. In both cases, the amount of calories was limited to the planned caloric intake. TPN was administered through a central venous catheter, with the use of 'all in one' ternary mixtures, by means of a continuous pump infusion. The TPN bag was replaced every 24 hours. EN was administered through a nasogastric or nasojejunal tube at the doctor's discretion and continuously through an infusion pump in accordance with a previously established protocol [9]. The systems used for EN administration were replaced at least once a day, and the feeding tube was flushed on a shift basis three times a day with 20 ml of distilled water. Malnutrition was assessed by means of the Subjective Global Assessment [10]. The calculated nutritional requirements were 25 kcal/kg per day (using the actual weight) with an intake of 1 to 1.5 g of protein/kg per day and a ratio of carbohydrates/fat of 60:40, in agreement with the recommendations published by the SEMICYUC (Spanish Society of Intensive Care) [11]. Fats used in the TPN group were long-chain triglyceride (LCT) or a physical admixture of medium-chain triglyceride (MCT)/LCT, according to the practice of each center. Enteral diets used in the EN group were always polymeric. Once the nutrition had been started, the following parameters were recorded: blood sugar and glucosuria every six hours; urea, creatinine, sodium, potassium, and chlorine every 24 hours; and a weekly analysis that included cholesterol, triglycerides, phosphorus, calcium, magnesium, and osmolarity. Liver function tests (total and direct bilirubin, aspartate aminotransferase, alanine aminotransferase, gamma-glutamyl-transferase, and alkaline phosphatase), prothrombin time, and international normalized ratio (INR) were recorded on admission and twice a week (on Tuesday and Friday). The withdrawal of artificial nutrition was defined as the definitive suppression of artificial nutrition, and suspension was defined as a temporary cancellation not longer than 24 hours.

### Definitions

The criteria used in this study to define the patterns of LD were the following: (a) cholestasis: alkaline phosphatase of more than 280 IU/l, gamma-glutamyl-transferase of more than 50 IU/l, or bilirubin of more than 1.2 mg/dl; (b) liver necrosis: aspartate aminotransferase of more than 40 IU/l, alanine aminotransferase of more than 42 IU/l, or INR of more than 1.4; and (c) mixed pattern: alkaline phosphatase of more than 280 IU/l, gamma-glutamyl-transferase of more than 50 IU/l, or bilirubin of more than 1.2 mg/dl, plus aspartate aminotransferase of more than 40 IU/l, alanine aminotransferase of more than 42 IU/l, or INR of more than 1.4. These boundaries represent a 10% increase of the normal values in the reference laboratories used. LD was diagnosed when any of the previously defined enzymatic alterations were present. The diagnosis of acalculous cholecystitis was based on clinical criteria and ultrasound. Liver biopsies were not carried out in this study.

### Statistical analysis

An intention-to-treat analysis was done for both types of nutrition, TPN and EN. The newly created database was centralized and managed by the main researchers. Any doubts about application of the protocol were discussed with the participants, and the main researchers made the final decision. Once the time of the study was over, the database was closed down. The analysis was blind to the type of nutrition used. The statistical analysis was performed using the SPSS v12 program (SPSS Inc, Chicago, Illinois, USA). The quantitative values were analyzed for normality. The values with normal distribution were compared using the Student's *t *test, and the others using non-parametric tests (Kruskall-Wallis test). The qualitative values were compared using Fisher's uncorrected chi-square test, and we calculated the relative risk with the confidence interval (CI) set at 95%. Statistical significance was set at *p *less than 0.05. The quantitative data were expressed as a median and interquartile (IQ) range, and the qualitative data were expressed in absolute values and percentages. The multivariate analysis for LD was carried out by means of a 'stepwise forward' logistical regression model with the most important demographic variables and those that reached statistical significance in the univariate analysis. Time free of LD was analyzed using the Kaplan-Meyer test.

## Results

### Description of the population

Three thousand four hundred and nine patients were admitted during the study. Seven hundred and fifty-six patients received nutrition in some form, whether TPN or EN, but 31 were excluded and 725 were studied (Table 1). Four hundred and eighty-eight were men and 237 were women. Three hundred and three patients (41.8%) received TPN and 422 (58.2%) received EN as the initial treatment. The patients who received TPN were older than those treated with EN (66 years, IQ range 48 to 73 years, versus 61 years, IQ range 45 to 71 years; *p *< 0.01) and mainly were women (38% versus 29%; *p *< 0.05). TPN was mostly used in surgical patients (175/264 versus 89/264; *p *< 0.001). Two hundred and eight patients had sepsis on admission; of these patients, 105 had septic shock. In both cases, TPN was used more frequently than EN. APACHE II score was higher in the group of patients who received EN (19, IQ range 13 to 23, versus 17, IQ range 12 to 22), without reaching statistical significance. More patients in the EN group required mechanical ventilation (91% versus 79%; *p *< 0.001). Also, ICU length of stay was longer in patients who received EN (12 days, IQ range 7 to 21 days, versus 8 days, IQ range 5 to 17 days; *p *< 0.001). Mortality, assessed 28 days after admission, showed no significant differences in either group (Table 2).

**Table 1:** Patient flow through the study

Patients admitted to the intensive care unit	3,409
Patients without artificial nutrition	2,653
Patients with artificial nutrition	756
Patients excluded	31
Patients studied	725
Patients on total parenteral nutrition	303
Patients also receiving enteral nutrition*	122
Patients on enteral nutrition	422
Patients also receiving total parenteral nutrition**	67

* Group of patients who received enteral nutrition after TPN

** Group of patients who received TPN after enteral nutrition

**Table 2:** Demographic data

	TPN	EN	Total	*p*
Number of patients	303	422	725	
Women	114 (38%)	123 (29%)	237 (33%)	0.02
Age in years	66 (48–73)	61 (45–71)	63 (47–72)	0.01
APACHE II score	17 (12–22)	19 (13–23)	18 (12–22)	0.08
MODS	5 (3–8)	5 (3–7)	5 (3–7)	0.95
Primary diagnosis				0.001
Gastrointestinal surgery	145	33	178	
Respiratory failure	21	112	133	
Stroke	22	103	125	
Cardiovascular	36	50	86	
Trauma	19	64	83	
Infections in non-immunosuppressed patients	18	22	40	
Infections in immunosuppressed patients	4	7	11	
Metabolic diseases	5	5	10	
Urology	4	6	10	
Hematology	7	2	9	
Poisoning	4	4	8	
Obstetrics/Gynecology	6	1	7	
AIDS	1	1	2	
Other	11	12	23	
Type of patients				0.001
Medical	105	257	362	
Surgical	175	89	264	
Trauma	23	76	99	
Sepsis on admission	122 (40%)	86 (20%)	208 (29%)	0.001
Septic shock on admission	70 (23%)	35 (8%)	105 (15%)	0.001
Patients on mechanical ventilation	239 (79%)	382 (91%)	621 (86%)	0.001
Days of mechanical ventilation	7 (2–16)	9 (4–17)	8 (3–16)	0.001
Intensive care unit length of stay in days	8 (5–17)	12 (7–21)	10 (6–20)	0.001
Hospital length of stay in days	25 (15–29)	25 (15–28)	25 (15–29)	0.6
Mortality at 28 days	85 (28.1%)	119 (28.2%)	204 (28%)	0.9

Parenthetical values indicate range or percentage. APACHE II, Acute Physiology and Chronic Health Evaluation II; EN, enteral nutrition; MODS, Multiple Organ Dysfunction Score; TPN, total parenteral nutrition.

The nutritional parameters were different in the two groups of patients. There was a significant statistical association between TPN and severe malnutrition (36% versus 15%; *p *< 0.001). The calculated energy requirements were similar in both groups as well as the days of artificial nutrition. Nutrition was started early after admission in both groups (median: 1 day, IQ range: 0 to 2 days), without differences between them. The duration of artificial nutrition was also similar in both groups (median: 9 days, IQ range: 5 to 8 days). One hundred and twenty-two patients assigned to the TPN group received EN when the gastrointestinal function recovered, and EN was stopped in 67 because they were unable to achieve the caloric requirements at day 3 or because they had EN-related complications. MCT/LCT admixtures were used in both groups when receiving TPN, without differences between them. Patients with EN received significantly fewer calories per kilogram on day 1 (20.8, IQ range 15.7 to 25, versus 22.9, IQ range 217.57 to 27.67; *p *< 0.01) and day 3 of the study (22.5, IQ range 17.65 to 26.87, versus 24.1, IQ range 20 to 29.45; *p *< 0.005) (Table 3).

**Table 3:** Nutritional parameters

	TPN	EN	Total	*p*
	303	422	725	
Weight	70 (63–80)	73 (65–80)	72 (65–80)	0.2
Nutritional status				0.001
Moderate malnutrition	76 (25%)	49 (12%)	125 (17%)	
Severe malnutrition	33 (11%)	14 (3%)	47 (7%)	
Energy requirements per kilogram	25 (23.29–29.37)	25 (23.76–30)	25 (23.64–29.74)	0.7
Patients receiving TPN	-	67		
Patients receiving EN	122	-		
Patients receiving MCT/LCT on TPN	186 (61%)	47 (71%)	233 (63%)	0.2
Days of artificial nutrition	8 (4–18)	10 (5–19)	9 (5–8)	0.2
Days on EN	1 (0–1)	9 (5–18)	6 (1–13)	0.001
Days on TPN	7 (3–11)	0 (0–1)	1 (0–7)	0.001
Starting time after ICU admission in days	1 (0–2)	1 (0–2)	1 0–2)	0.6
Prescribed caloric intake per kilogram on day 1	24.65 (18.77–28.57)	23.53 (20.00–26.67)	24 (19.3–27.64)	0.09
Administered caloric intake per kilogram on day 1	22.92 (17.57–27.67)	20.8 (15.72–25)	21.43 (16.36–26.28)	0.01
Prescribed caloric intake per kilogram on day 3	25 (21.25–30)	25 (21.25–28.57)	25 (21.25–29.36)	0.3
Administered caloric intake per kilogram on day 3	24.17 (20–29.45)	22.5 (17.65–26.87)	23.14 (18.69–27.99)	0.003
Prescribed caloric intake per kilogram on day 7	25.84 (22.22–29.94)	25.35 (21.43–30)	25.66 (21.43–30)	0.6
Administered caloric intake per kilogram on day 7	24.72 (20–29.46)	24.06 (19.63–28.57)	24.31 (19.76–28.61)	0.2

Parenthetical values indicate range or percentage. EN, enteral nutrition; ICU, intensive care unit; MCT/LCT, medium-chain triglyceride/long-chain triglyceride; TPN, total parenteral nutrition.

### LD and artificial nutrition

One hundred and sixty-six patients (23%) had LD. There was a significant statistical association between the appearance of LD and age (*p *< 0.01), the MODS score (*p *< 0.001), in surgical (35%) and trauma patients (41%) (*p *< 0.03), if they had sepsis (*p *< 0.001) or septic shock on admission (*p *< 0.02), and in patients who were mechanically ventilated (*p *< 0.02). The stay in the ICU (16 days, IQ range 8 to 28 days, versus 9 days, IQ range 5 to 17 days; *p *< 0.001) and in the hospital (28 days, IQ range 17 to 29 days, versus 23 days, IQ range 14 to 28 days; *p *< 0.01) was longer in the group with LD. No difference in mortality was shown between the two groups (Table 4). The patients with LD were less nourished (33% versus 21%; *p *< 0.01) and were treated mostly with TPN (30% versus 18%; *p *< 0.001) for more days (13 days, IQ range 8 to 25, versus 8 days, IQ range 4 to 16 days; *p *< 0.001). Patients fed early had significantly less LD. The use of MCT/LCT admixtures was similar in patients with or without LD, but the calculated energy requirements were higher (25.54 kcal/kg per day, IQ range 24.49 to 30 kcal/kg per day, versus 25 kcal/kg per day, IQ range 23.33 to 29.41 kcal/kg per day; *p *< 0.05) (Table 5).

**Table 4:** Demographic data in patients with and without liver dysfunction

	With liver dysfunction	Without liver dysfunction	Total	*p*
Number of patients	166 (23%)	559 (77%)	725	
Women	54 (33%)	183 (33%)	237 (33%)	0.9
Age in years	63 (47–72)	63 (44–73)	63 (47–72)	0.8
APACHE II score	18 (14–23)	18 (12–22)	18 (12–22)	0.2
MODS	6 (4–8)	5 (3–7)	5 (3–7)	0.001
Primary diagnosis				0.4
Gastrointestinal surgery	52	126	178	
Respiratory failure	26	107	133	
Stroke	27	98	125	
Cardiovascular	14	72	86	
Trauma	16	67	83	
Infections in non-immunosuppressed patients	12	28	40	
Infections in immunosuppressed patients	3	8	11	
Metabolic diseases	1	9	10	
Urology	3	7	10	
Hematology	1	8	9	
Poisoning	2	6	8	
Obstetrics/Gynecology	3	4	7	
AIDS	1	1	2	
Other	5	18	23	
Type of patients				0.03
Medical	68	294	362	
Surgical	69	195	264	
Trauma	29	70	99	
Sepsis on admission	68 (41%)	140 25%)	208 (29%)	0.001
Septic shock on admission	33 (20%)	72 (13%)	105 (15%)	0.02
Patients on mechanical ventilation	152 (92%)	469 (84%)	621 (86%)	0.01
Days of mechanical ventilation	13 (6–24)	7 (3–14)	8 (3–16)	0.001
Intensive care unit length of stay in days	16 (8–28)	9 (5–17)	10 (6–20)	0.001
Hospital length of stay in days	28 (17–29)	23 (14–28)	25 (15–29)	0.01
Mortality at 28 days	47 (28.3%)	157 (28.1%)	204 (28%)	0.9

Parenthetical values indicate range or percentage. APACHE II, Acute Physiology and Chronic Health Evaluation II; MODS, Multiple Organ Dysfunction Score.

**Table 5:** Nutritional parameters in patients with and without liver dysfunction

	With liver dysfunction	Without liver dysfunction	Total	*p*
	166	559	725	
Weight in kilograms	75 (65–80)	70 (65–80)	72 (65–80)	0.3
Nutritional status				0.01
Moderate malnutrition	41 (25%)	84 (15%)	125 (17%)	
Severe malnutrition	14 (8%)	33 (6%)	47 (7%)	
Energy requirements per kilogram	25.54 (24.49–30)	25 (23.33–29.41)	25 (23.64–29.74)	0.04
Type of nutrition				0.001
Enteral	75 (18%)	347 (82%)	422	
Parenteral	91 (30%)	212 (70%)	303	
Patients receiving MCT/LCT on TPN	75	158	233	0.2
Days on artificial nutrition	13 (8–25)	8 (4–16)	9 (5–8)	0.001
Days on EN	7 (1–17)	6 (0–12)	6 (1–13)	0.2
Days on TPN	5 (0–12)	0 (0–5)	1 (0–7)	0.001
Starting time after ICU admission in days	1 (0.5–2)	1(0–2)	1 (0.2–2)	0.03
Prescribed caloric intake per kilogram on day 1	25 (20.92–29.34)	23.53 (18.75–27.27)	24 (19.3–27.64)	0.01
Administered caloric intake per kilogram on day 1	22.30 (16.88–26.67)	21.43 (16.25–26.15)	21.43 (16.36–26.28)	0.3
Prescribed caloric intake per kilogram on day 3	25 (21.67–30)	24.69 (21.18–28.57)	25 (21.25–29.36)	0.07
Administered caloric intake per kilogram on day 3	23.67 (18.79–28.92)	23.07 (18.70–27.54)	23.14 (18.69–27.99)	0.4
Prescribed caloric intake per kilogram on day 7	26.67 (23.29–30)	25 (20.93–30)	25.66 (21.43–30)	0.06
Administered caloric intake per kilogram on day 7	25 (19.46–29.79)	24.01 (19.85–28.33)	24.31 (19.76–28.61)	0.3

Parenthetical values indicate range or percentage. EN, enteral nutrition; ICU, intensive care unit; MCT/LCT, medium-chain triglyceride/long-chain triglyceride; TPN, total parenteral nutrition.

### LD, TPN, and type of patients

**Figure 1:**
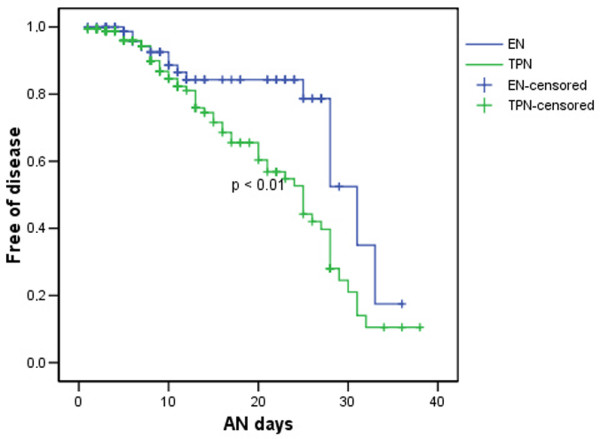
Time free of liver dysfunction in surgical patients treated with Enteral Nutrition or Total Parenteral Nutrition. EN, enteral nutrition; TPN, total parenteral nutrition; AN days, days on artifical nutrition

In the univariate analysis, 91 patients treated with TPN developed some form of LD but only 75 in the EN group did (odds ratio [OR] 1.7, 95% CI 1.3 to 2.2) (Table 6). Surgical patients (31% versus 16%; OR 1.8, 95% CI 1.02 to 3.1) and trauma patients (52% versus 23%; OR 2.1, 95% CI 1.1 to 4) treated with TPN had more LD. This association was maintained for all types of LD: cholestasis (OR 1.7, 95% CI 1.04 to 2.9), liver necrosis (OR 1.95, 95% CI 1.1 to 3.42), and mixed pattern (OR 1.8, 95% CI 1.3 to 2.6). The patients with sepsis and TPN showed a higher incidence of LD than the group treated with EN (39% versus 24%; OR 1.6, 95% CI 1.02 to 2.4), although no type of LD was greater in either group. When looking at the time free of LD, EN increased the time free of disease in surgical patients only in the Kaplan-Meyer survival test (Figure 1). Only three patients were diagnosed with acalculous cholecystitis.

**Table 6:** Incidence of liver dysfunction

	TPN	EN	Total	*p*	OR (95% CI)
Overall patients	303	422	725		
Liver dysfunction	91 (30%)	75 (18%)	166 (23%)	0.001	1.7 (1.3–2.2)
Cholestasis	31 (10%)	25 (6%)	56 (8%)	0.03	1.7 (1.04–2.9)
Hepatic necrosis	28 (9%)	20 (5%)	48 (7%)	0.02	1.95 (1.1–3.4)
Mixed pattern	56 (19%)	43 (10%)	99 (14%)	0.001	1.8 (1.3–2.6)
Acalculous cholecystitis	1	2	3		
Type of patients and liver dysfunction	91	75	166		
Medical	24 (23%)	44 (17%)	68 (19%)	0.3	1.2 (0.8–1.7)
Surgical	55 (31%)	14 (16%)	69 (26%)	0.03	1.8 (1.02–3.1)
Trauma	12 (52%)	17 (23%)	29 (37%)	0.02	2.1 (1.1–4)
Septic patients	122	86	208		
Liver dysfunction	47 (39%)	21 (24%)	68 (33%)	0.03	1.6 (1.02–2.4)
Cholestasis	17 (14%)	6 (7%)	23 (11%)	0.1	1.9 (0.8–4.9)
Hepatic necrosis	10 (8%)	3 (4%)	13 (6%)	0.2	2.4 (0.7–8.2)
Mixed pattern	30 (25%)	14 (16%)	44 (21%)	0.1	1.5 (0.9–2.7)
Non-septic patients	181	336	517		
Liver dysfunction	44 (24%)	54 (16%)	98 (19%)	0.02	1.5 (1.06–2.2)
Cholestasis	14 (8%)	19 (6%)	33 (6%)	0.4	1.4 (0.7–2.7)
Hepatic necrosis	18 (10%)	17 (5%)	35 (7%)	0.04	2 (1.03–3.7)
Mixed pattern	26 (14%)	29 (9%)	55 (11%)	0.04	1.7 (1.01–2.7)

CI, confidence interval; EN, enteral nutrition; OR, odds ratio; TPN, total parenteral nutrition.

### Multivariate analysis

The risk factors associated with LD in the multivariate analysis were TPN (OR 1.96, 95% CI 1.3 to 2.97, *p *< 0.001), the early use of artificial nutrition (TPN or EN) the first day after admission (OR 0.6, 95% CI 0.4 to 0.9, *p *< 0.01), MODS (OR 1.1, 95% CI 1.04 to 1.2, *p *< 0.001), and the diagnosis of sepsis on admission (OR 1.76, 95% CI 1.08 to 2.9, *p *< 0.02). The rest of the variables analyzed, such as age, gender, APACHE II score, septic shock on admission, medical patients, surgical patients, mechanical ventilation, the use of MCT/LCT admixtures, or severe malnutrition, did not reach statistical significance in the logistical regression model (Table 7).

**Table 7:** Logistic regression analysis for liver dysfunction

	OR	95% CI	*p*
TPN	1.97	1.3–3	0.002
MODS	1.1	1.04–1.2	0.003
Early artificial nutrition (first day)	0.6	0.4–0.9	0.01
Energy requirements < 25 kcal/kg per day	0.62	0.41–0.94	0.03
Sepsis	1.76	1.08–2.9	0.05
Mechanical ventilation	0.5	0.3–1.07	0.05
Medical patient	0.6	0.3–1.02	0.06
MCT on TPN	1.4	0.9–2.2	0.09
APACHE II score	0.98	0.94–1.01	0.2
Surgical patient	0.6	0.3–1.2	0.2
Severe malnutrition	0.8	0.39–1.7	0.6
Septic shock	1.2	0.6–2.2	0.6
Gender (women)	0.98	0.65–1.48	0.9
Age	0.99	0.98–1.01	0.9

APACHE II, Acute Physiology and Chronic Health Evaluation II; CI, confidence interval; MCT, medium-chain triglyceride; MODS, Multiple Organ Dysfunction Score; OR, odds ratio; TPN, total parenteral nutrition.

## Discussion

Our study shows that the incidence of LD associated with artificial nutrition in seriously ill patients is low (23%) and is more frequent in patients who received TPN, with sepsis on admission, and when the planned calculated caloric intake was higher than 25 kcal/kg per day. LD is a widely recognized complication associated with the use of artificial nutrition, particularly TPN, with an incidence of between 25% and 100% [12,13]. Acalculous cholecystitis was diagnosed in only three patients who received TPN, with an incidence of close to the 4% published elsewhere [12].

Multiple factors are related to LD associated with TPN, linked to the type of formulation or the appearance of nutritional deficiencies with the use of TPN [13-16]. Some of these factors are shortage of essential fatty acids [17,18], excessive caloric intake [19], imbalance in the composition of the amino acids [20] or of the non-protein substrates [21], fat deposit in the liver [22], a caloric intake based exclusively on fats [23], a cholestatic effect of the amino acids [24], the absence of choline [25], production of endotoxins and lithocholic acid due to intestinal bacterial overgrowth [26], shortage of carnitine [27], or the absence of enteral nutritional intake [28,29].

However, few studies examine the risk factors attributable to the clinical state of the patient. The aims of this study were to identify the relationship between the appearance of LD and the use of artificial nutrition and to identify the contributing factors specific to the critically ill patient (severity scores, associated co-morbidity such as sepsis, and mechanical ventilation) which can act as confusion factors. Many studies have demonstrated the superiority of EN over TPN, both in surgical patients [30-33] and in patients admitted to the ICU [34,35]. Our results show that patients who received EN had a lower incidence of LD. Most patients who received EN were medical, were more in need of mechanical ventilation, and had a longer stay in the ICU but showed less LD (18% in the EN group versus 30% in the TPN group). This result is strong enough because we have performed an 'intention to treat analysis,' and the 16% of the patients on EN also received TPN. We have found that other factors, such as previous gastrointestinal surgery or sepsis on admission, can explain the greater incidence of LD shown in the results of our study and in other studies [36,37].

Our study shows that cholestasis and the mixed pattern are the two most frequent types of LD. The elevations of serum transaminases, alkaline phosphatase, and bilirubin are the changes most often associated with the use of TPN [38,39]. Although the increase of serum transaminases usually takes place in the first two or three weeks of TPN, it is unusual to observe a significant increase of bilirubin in this period, at least in adult patients [40-42]. In many cases, these enzymatic alterations are mild and transient, even without the interruption of TPN, and only occasionally lead to liver steatosis. Fat infiltration and intrahepatic cholestasis are the typical findings in these patients [28,43,44]. The progress of this LD is generally self-limiting but can lead to liver failure in a minority of patients [38,39,44]. Liver biopsies showed that the predominant finding in patients with enzymatic alterations is liver steatosis [3,11]. When biopsies are carried out in different periods of time, steatosis is an early and sometimes transient phenomenon, whereas cholestasis is a later finding and generally persists during the TPN. Nevertheless, there are contradictory data between an abnormal level of the hepatic enzymes and steatosis or cholestasis [43,44]. Interestingly, our data show that the early use of artificial nutrition, TPN or EN, can delay the appearance of any type of LD and can avoid permanent liver damage in these patients.

Another factor that could contribute to the low incidence of LD found in our group is related to the composition of the TPN. There are studies that emphasize the effect of overfeeding on the hepatic metabolism [45-47] or suggest that a lipid mixture containing MCTs (MCT/LCT) could decrease the risk of steatosis or liver cholestasis [48]. Our results do not confirm this protective effect of the MCT/LCT lipid admixture. The energy requirements of our patients were calculated at 25 kcal/kg per day. We have noted a significant difference in the administered calories in the TPN group compared with the EN group on the first and third days of follow-up, as well as a larger energy intake administered the first day of nutrition in the group of patients who developed LD. The carbohydrate/fat ratio (60:40) that we used in this study seems to be safe and can prevent the abnormalities in liver tests [49].

## Conclusion

Our results show that the patients who developed LD have a characteristic profile in the multivariate analysis. They had a higher MODS on admission, they were septic, and they were treated with TPN. The assessment of multiple organ dysfunction includes among its parameters an LD based on high levels of bilirubin, so this association should be expected. The liver is the key organ in the starting and development of multiple organ dysfunction in the septic patient and plays an essential role by clearing endotoxins, bacteria, and derived vasoactive substances. Sepsis and inflammation can increase the production of cytokines, which are potent inhibitors of bile secretion, and the consequent development of cholestasis that can be enhanced by TPN. Although the negative effects that both TPN and sepsis exert on hepatic metabolism have previously been studied independently, this study shows that there is a greater effect when both conditions, TPN and sepsis, are present. Also, early artificial nutrition seems to exert a beneficial effect. Notwithstanding prevention and treatment measures, the presence of sepsis and multiple organ failure should compel to clinicians to strictly control the caloric intake of seriously ill patients, start artificial nutrition early, and frequently monitor their liver function.

## Key messages

• Critically ill patients on artificial nutrition who developed LD have a characteristic profile: they had a higher MODS score on admission, they were septic, and they were treated with TPN and nutrition was started later.

• Sepsis and the use of TPN are the most important conditions that increase the incidence of liver failure.

• Cholestasis and the mixed pattern are the most frequent patterns of LD.

• Acalculous cholecystitis is an uncommon finding in our patients.

## Abbreviations

APACHE II = Acute Physiology and Chronic Health Evaluation II; CI = confidence interval; EN = enteral nutrition; ICU = intensive care unit; INR = international normalized ratio; IQ = interquartile; LCT = long-chain triglyceride; LD = liver dysfunction; MCT = medium-chain triglyceride; MODS = Multiple Organ Dysfunction Score; OR = odds ratio; TPN = total parenteral nutrition.

## Competing interests

B. Braun Medical S.A., Cta de Tarrasa 121, 08191 Barcelona, Spain has financially supported the data acquisition, but without access to the database or results, and will support the article-processing charge. TG is a member (vice-coordinator) of the Spanish Working Group on Metabolism and Nutrition (section of the Spanish Society of Critical Care). ABo is the coordinator of the Spanish Working Group on Metabolism and Nutrition (section of the Spanish Society of Critical Care). The other authors declare that they have no competing interests.

## Authors' contributions

TG and ABo conceived the study, participated in its design and coordination, and helped to draft the manuscript. TG performed the statistical analysis. MR and DM were involved in drafting the manuscript or revising it critically for important intellectual content. ABl, MF, JAA, and JCM participated in the design of the study and they coordinated the meetings with the participants. AG and AM have given final approval of the version to be published. All authors read and approved the final manuscript.
